# Development of a geometry‐based respiratory motion– simulating patient model for radiation treatment dosimetry

**DOI:** 10.1120/jacmp.v9i1.2700

**Published:** 2008-01-21

**Authors:** Juying Zhang, X. George Xu, Chengyu Shi, Martin Fuss

**Affiliations:** ^1^ Nuclear Engineering and Engineering Physics Rensselaer Polytechnic Institute Troy New York; ^2^ Radiation Oncology Cancer Therapy and Research Center San Antonio Texas; ^3^ Department of Radiation Medicine Oregon Health and Science University Portland Oregon U.S.A.

**Keywords:** 4D, phantom, Monte Carlo, anatomic model, EGS4

## Abstract

Temporal and spatial anatomic changes caused by respiration during radiation treatment delivery can lead to discrepancies between prescribed and actual radiation doses. The present paper documents a study to construct a respiratory‐motion‐simulating, four‐dimensional (4D) anatomic and dosimetry model for the study of the dosimetric effects of organ motion for various radiation treatment plans and delivery strategies. The non‐uniform rational B‐splines (NURBS) method has already been used to reconstruct a three‐dimensional (3D) VIP‐Man (“visible photographic man”) model that can reflect the deformation of organs during respiration by using time‐dependent equations to manipulate surface control points. The EGS4 (Electron Gamma Shower, version 4) Monte Carlo code is then used to apply the 4D model to dose simulation. We simulated two radiation therapy delivery scenarios: gating treatment and 4D image‐guided treatment. For each delivery scenario, we developed one conformal plan and one intensity‐modulated radiation therapy plan. A lesion in the left lung was modeled to investigate the effect of respiratory motion on radiation dose distributions. Based on target dose–volume histograms, the importance of using accurate gating to improve the dose distribution is demonstrated. The results also suggest that, during 4D image‐guided treatment delivery, monitoring of the patient's breathing pattern is critical. This study demonstrates the potential of using a “standard” motion‐simulating patient model for 4D treatment planning and motion management.

PACS numbers: 87.53.Bn, 87.53.Kn, 87.53.Tf, 87.53.Wz, 87.57.Gg, 89.80.+h

## I. INTRODUCTION

External‐beam radiation therapy for malignant tumors aims to sterilize tumor clones while preserving the organ from which the tumor originates and the surrounding normal tissues. Since the late 1980s, methods to target the tumor treatment volume have gradually evolved in step with advancing medical imaging technologies capable of three‐dimensional (3D) and even four‐dimensional (4D) volume imaging. Current technologies, such as intensity‐modulated radiation therapy (IMRT) and image‐guided radiation therapy permit more accurate and conformal dose planning and delivery to target volumes.^(^ ^1^ ^–^ ^7^ ^)^ Ideally, during planning and delivery, the precise location and shape of the tumor relative to the beam geometry defined by a medical linear accelerator would be known.^(7)^ Although the foregoing assumption is acceptable for tumors in the brain or near the body surface, a favorable rigid‐body relationship does not exist in anatomic sites such as the thoracic cavity and the abdomen, predominantly because of respiratory motion. When the tumor and its bearing organ undergo motion and deformation during radiation therapy delivery, significant discrepancies between the planned and delivered radiation doses have been reported.^(^ ^1^ ^–^ ^9^ ^)^

Management of organ motion has become one of the most difficult challenges in radiation oncology. Anatomic models that describe geometric changes in organs over time offer a unique opportunity to tackle various issues related to dose planning and monitoring and to radiation delivery. To account for temporal and spatial anatomic changes during radiation treatment, a means to specify organ and tumor motion in real time is desirable. This information needs to be incorporated into the radiation therapy planning process and to accurately reflect target and normal anatomic motion during subsequent radiation dose deliveries. This goal has only recently been formulated in the field of radiation oncology, and it has not yet been clinically implemented.^(5)^ With the advent of so‐called 4D computed tomography (CT), accounting for target changes is becoming possible in routine applications.^(10)^

Developments in 4D treatment planning^(^ ^11^ ^–^ ^15^ ^)^ and 4D radiation dose delivery^(^ ^5^ ^,^ ^16^ ^,^ ^17^ ^)^ have been reported by many researchers. However, the clinical implementation of 4D CT–based treatment planning is currently still in its infancy, with a key emphasis on defining the motion envelope of a target volume in terms of an internal target volume (ITV). In such an approach, a single free‐breathing CT dataset is replaced by a series of up to 10 CT datasets sampled during various phases of a respiratory cycle.^(^ ^10^ ^,^ ^14^ ^)^ However, evidence suggests that the true anatomic relationships between 4D CT image frames are uncertain, and current image‐processing methods suffer from considerable artifacts.^(^ ^18^ ^,^ ^19^ ^)^ In addition, because the field size of a typical CT scan is limited, the interlinked motions and deformations involving several organs (such as heart, lungs, liver, etc.) are often difficult to analyze.

We therefore hypothesized that a detailed respiratory‐motion‐simulating 4D patient model containing realistic anatomy and physiology coupled with Monte Carlo simulation capabilities would offer unique insights into strategies for solving a number of the foregoing issues. The present paper describes our preliminary efforts in developing a geometry‐based respiratory‐motion‐simulating model in which Monte Carlo simulations could be used to assess dose distributions. The general modeling approach is to use time‐dependent surface equations that follow respiratory motion patterns to deform various organs in a series of 3D geometries. The advantage of this approach is that it creates a “standardized” patient model whose interlinked organ motion dynamic is fully controlled in the modeling process. This anatomic‐model‐based approach can complement information obtained from patient‐specific 4D CT data.

## II. MATERIALS AND METHODS

**Figure 1:**
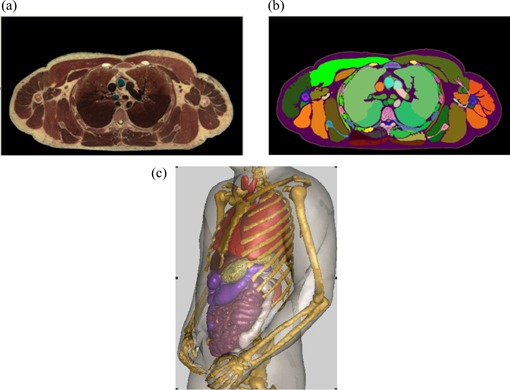
(a) A slice of the original Visible Human cryosectioned color image for the chest region. (b) The same slice after segmentation and labeling. (c) A three‐dimensional rendering of the internal organs of the reconstructed VIP‐Man model.

Modeling of patient anatomy for radiation dosimetry has a long history. Over the years, technologies, especially medical imaging and modern computers, have allowed whole‐body computational patient models to evolve into three types in terms of geometric definition:

- The first type of whole‐body model is the equation‐based stylized model whose organs are delineated by a combination of surface equations of very simple geometries. The earliest such models were developed at Oak Ridge National Laboratory for the Medical Internal Radiation Dosimetry Committee of the Society of Nuclear Medicine in the 1970s.^(^ ^20^ ^,^ ^21^ ^)^ Three media with distinct densities were specified: bone, soft tissue, and lung. Models of this kind were analytically defined in three anatomic sections: an elliptical cylinder representing the arm, torso, and hips; truncated elliptical cones representing the legs and feet; and an elliptical cylinder representing the head and neck. The formulation of the geometries was based on reference data and the need to accommodate computational abilities at the time. This original model was later revised to yield a family of models of both sexes and various ages.^(22)^ Minor revisions have been reported since the original development, and this type of model dominated applications between the 1970s and the 1990s.
- The second type is the image‐based tomographic model, consisting of organs that are defined from tomographic images of real individuals. These computational models took advantage of the 3D tomographic imaging that became available starting near the end of the 1980s. Such models contain large arrays of voxels identified in terms of tissue type (for example, soft tissue, hard bone, and air) and identification (for example, lungs, liver, and skin). Tomographic images reveal internal structures realistically, but time‐consuming segmentation and classification are necessary if the models are to be used for radiation transport calculations in a Monte Carlo code. Nearly 30 such tomographic models representing various ages and both sexes have been reported to date.^(23)^ The International Commission on Radiological Protection has recommended a programmatic shift from stylized models to tomographic models in radiation protection dosimetry.^(^ ^24^ ^–^ ^29^ ^)^ In 2000, we reported the development of an adult male model named VIP‐Man (“visible photographic man”).^(30)^ That model was based on anatomic color images of the Visible Man from the Visible Human Project.^(^ ^31^ ^,^ ^32^ ^)^ The original image resolution of the Visible Man was 0.33×0.33 mm, and the slice thickness was 1 mm, which allowed for small and radiosensitive structures to be identified and modeled, including skin, eye lenses, and red bone marrow.^(30)^ Fig. 1(a) shows a slice of the original Visible Human cryosectioned color image for the chest region. Fig. 1(b) shows the same slice after segmentation and labeling. Fig. 1(c) is a 3D surface rending of the VIP‐Man internal organs. The VIP‐Man model has been successfully used for Monte Carlo radiation studies of organ doses for photons, electrons, neutrons, and protons.^(^ ^33^ ^–^ ^44^ ^)^
- The third type of model uses advanced geometry‐based surface primitives based on tomographic image data to describe each organ. Voxel‐based tomographic models, although realistic, are known to be difficult for defining the deformation of organs in real time. The large number of boundary crossings defined by the voxels significantly increases computation time during radiation transport in Monte Carlo simulations. It became clear that the voxel data could be transformed to surface equations [such as non‐uniform rational B‐splines (NURBS) surfaces^(45)^], so that the organ shapes are anatomically realistic as compared with stylized models but are at the same time convenient for modeling anatomic deformation attributable to organ motion. This approach can use patient‐specific images and image registration as necessary to adjust the boundaries of an organ to the desired shape and volume. To date, this type of anatomic modeling has been limited. Examples include the 4D NURBS‐based cardiac–torso (NCAT) model by Segars et al.^(^ ^46^ ^–^ ^48^ ^)^ that used the CT images of the Visible Human Project, and a more recent work by Xu et al.^(49)^ that used the VIP‐Man model originally developed from the color anatomic images of the Visible Human Project. The NCAT model has been used primarily in nuclear medicine; the VIP‐Man model is used in radiation dosimetry. This type of modeling approach has been adopted by a number of groups since 2005.^(50)^

Literature reviews of the three model types suggest the feasibility of adopting the NURBS‐based approaches to create “standard” motion‐stimulating 4D patient models for the dosimetric study of respiratory motion management for radiation treatment. The detailed anatomic information available from the previously developed VIP‐Man model can be coupled with clinical respiration data to derive such 4D models for radiation dosimetry.

A 4D motion‐simulating anatomic model is a series of 3D tomographic models whose shape, size, and location change according to specific respiratory motion patterns. Our modeling work followed these specific steps:

- Conversion from voxel data to surface definition
- Deformation of the organ surfaces
- Revoxelization of the 4D model for each respiratory phase
- Implementation of the 4D model into a Monte Carlo code for dose calculations
- Final integration to obtain dose distributions for the lesion

In the subsections that follow, each of these steps is described in more detail.

### A. Conversion from voxel data to surface definition

The VIP‐Man whole‐body model contains voxels at a size of 0.33×0.33×1 mm. In a previous study, manual and automatic procedures were used to carefully segment the organs.^(30)^ Fig. 1(c) shows the internal organs of the 3D VIP‐Man model as adopted for the present study. Organs, each originally defined as a group of voxels, were converted into polygon surface models to extract the anatomic features. Polygon models were then translated into NURBS surfaces.^(45)^ Organ surfaces defined using NURBS can easily be deformed by changing control points, as first demonstrated by Segars and colleagues^(^ ^46^ ^–^ ^48^ ^)^ and later by Xu and Shi.^(49)^ The NURBS approach has also been used to manually adjust shape and size for a series of pediatric models.^(50)^

For the present study, a commercial program called Rhinoceros (McNeel North America, Seattle, WA) was used to create, edit, analyze, and translate NURBS curves, surfaces, and solids. A free software program called vtkEditor (homes.esat.kuleuven.be/~open3d/) was used to change the VIP‐Man organ files into a format readable by Rhinoceros. Once the organs were imported into Rhinoceros, organ contours were regenerated and lofted into 3D NURBS surfaces. Thus far, this process has been performed for lungs, heart, skin, rib cage, spine, kidneys, stomach, spleen, and liver. Additional organs and tissues can be generated using the same method.

After the NURBS files were generated, control points for each organ were obtained by exporting the NURBS organ file to a text file. These control points contain basic anatomic features of the original 3D VIP‐Man model.

### B. Deformation of the organ surfaces

To simulate time‐dependent deformation caused by respiratory motion, the control points for each of the interlinked organs were transformed using a rigid motion

(1) $$C_{new}=S\times R\times C_{old}+T,$$

in which $C_{\text{old}}$ is a 3×N matrix of the original control points (*N* being the number of control points in the given organ), $C_{\text{new}}$ is the 3×N matrix of translated points, *S* is a scalar matrix, *R* is a 3×3 matrix that defines rotation, and *T* is 3×N matrix that defines the translation. By using parameters that are functions of time, *t*, in matrixes *S, R*, and *T*, the control points are extended from the original 3D space into 4D. In the present study, the respiratory motion patterns were modeled using clinical data described previously.^(46)^ The rotation angle of the rib cage around its intersection with the spine is defined as a piecewise linear function with respect to time, *t*,

(2) $$\theta =\{{\begin{array}{ll} \begin{array}{ll} \Delta \theta \times t & 0\leq t<2 \end{array}\\ \frac{2}{3}\times \Delta \theta \times (5-t) & 2\leq t<5 \end{array}}_{,}$$

in which we selected the angular step, Δθ, to be 2.5 degrees, and the entire respiration cycle to last 5 seconds. The first 2 seconds were used for inspiration, and the last 3 seconds, for expiration. The positions of the sternum and skin were defined according to the positions of the rib tips. The lungs were modeled by referring to the position of the fifth rib in the transverse direction and the top of the liver in the longitudinal direction. For other organs, such as liver, stomach, heart, kidneys, and a lesion in the left lung, we first created a normalized motion curve,

(3) $$x=\{{\begin{array}{ll} \frac{1}{2}\left(1-\cos(\frac{\pi }{2}\times t)\right) & 0\leq t<2\\ \frac{1}{2}\left(1-\cos(\frac{\pi }{3}\times (5-t))\right) & 2\leq t<5 \end{array}}_{,}$$

in which *x* is the normalized motion distance, and *t* is the time. The magnitude of the organ motion was then controlled by multiplying various amplifying factors in the *x, y, z* directions respectively. We used MATLAB 6.5 (The Mathworks, Natick, MA) to develop a software program to automate the calculations and a NURBS Toolbox (www.aria.uklinux.net/nurbs.php3) for MATLAB to perform the final integration of the 4D VIP‐Man model.

### C. Revoxelization of the 4D model for each respiratory phase

For calculation of the radiation dose distributions in each of the respiratory phases, the 4D model was treated as a combination of a series of 3D models representing the anatomy at various respiratory phases. Once the 4D VIP‐Man model had been constructed, each of the 3D models was re‐created from the 4D model for Monte Carlo dose calculations. That process was accomplished by converting the NURBS surfaces back to voxels for each given respiratory phase. The control points for an organ were saved in a 3D matrix, and the contours were calculated by specifying the cutting plane coordinate in the MATLAB program. A total of eight respiration phases, each consisting of 70 two‐dimensional (2D) slices, were sampled and revoxelized to represent the entire respiratory cycle: peak exhale, early inhale, middle inhale, late inhale, peak inhale, early exhale, middle exhale, and late exhale. The resolution for each of the revoxelized models is 2.1×1.2×6.0 mm. Finer resolutions can be obtained if the 2D slice size and cutting plane number are increased.

### D. Implementation of the 4D model into a Monte Carlo code for dose calculations

Monte Carlo simulations of the 4D model were performed using EGS4 (Electron Gamma Shower, version 4).^(51)^ To study the dose distributions in the lesion, two radiation treatment delivery scenarios were simulated. One delivery scenario represented a “gating treatment, gating being the method currently in use for the treatment of lung cancer at many centers. In that method, the radiation beam is turned on only when the patient's respiration reaches a particular breathing phase (usually peak inhale or peak exhale). The other delivery scenario represented a 4D image‐guided treatment, a method that has not yet been clinically adopted. In that method, we assumed that the radiation beam closely follows the moving lesion in each of the respiratory phases, and that the beam is always turned on during treatment delivery. For each treatment delivery scenario, one conformal plan and one IMRT plan were considered in the dose calculations.

**Figure 2:**
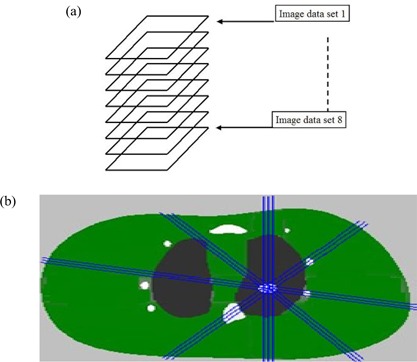
(a) Implementation of the four‐dimensional model in the Monte Carlo code by piling of the eight sets of images. (b) Geometry setting for intensity‐modulated radiotherapy simulation.

The 4D model (consisting of 8 respiratory sets) was implemented into the EGS4 code as a single calculation [Fig. 2(a)]. By changing the coordinates of the sampling sources according to time, *t*, we calculated the “gating” and the “4D treatment” to the lesion target specified in the 4D model. The energy of the photon beams was assumed to be 6 MV, and the irradiation geometry was assumed to be anterior–posterior (AP), posterior–anterior (PA), right lateral (RLAT), and left lateral (LLAT). The lesion was assumed to be a sphere with a radius of 5 mm. The statistical uncertainty in the Monte Carlo calculations was controlled to be less than 1 % for $1\times 10^{7}$ photons. The cutoff energies in the EGS4 code were set to be 100 keV for electrons and 10 keV for photons. The simulations were performed on a personal computer with a 2.66 GHz processor, and the simulation time was about 3 hours for each of the respiratory phases.

The IMRT photon intensity was first optimized using the ADAC ${\text{Pinnacle}}^{3}$ treatment planning system (Philips Medical Systems, Bothell, WA). After optimization, the IMRT radiation source is expressed in terms of the opening density matrix (ODM). The ODM consists of multi‐segment multileaf collimator (MLC) fields.

The ODM was exported in a binary file, and the intensity was sampled from the file. The beam angles were selected to be 80 degrees, 140 degrees, 180 degrees, and 220 degrees, as shown in Fig. 2(b). The beam energy was the same as that used in the EGS4 simulations. The source‐axis distance (SAD) was 100 cm, and the final isocenter was the center of the lesion inside the left lung.

### E. Final integration to obtain the lesion dose distributions

To calculate the final radiation dose distributions to the moving lesion, individual doses from the eight respiratory motion phases had to be totaled. The target voxels corresponding to each motion phase needed to be tracked during the respiratory motion within a universal reference coordinate. The registration process was based a reversed transformation of equation 1:

(4) $$C_{old}^{\prime }=\frac{1}{S}\times R^{-1}\left(C_{new}^{\prime }-T\right).$$

For each respiratory phase, the control points for an organ are first converted into physical coordinates. Equation 4 is then used to transform the physical coordinates into the physical coordinates of the reference phase. The corresponding voxels in the reference phase can be determined by converting the physical coordinates into voxel numbers. Finally, the total dose and average dose can be derived. In the present study, because the details of the relationships of how organs deform are known during the modeling process, motion patterns and the image registration process were facilitated.

## III. RESULTS AND DISCUSSION

**Figure 3:**
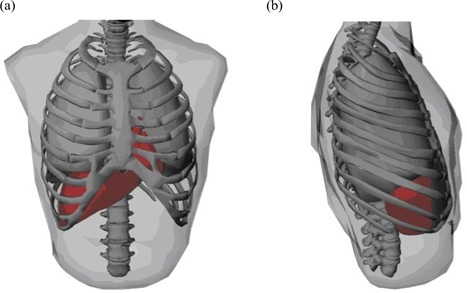
(a) Front view and (b) side view of the reconstructed non‐uniform rational B‐spline surfaces in phase 1 (middle inhale phase).

Using the detailed procedures described so far, we developed and tested a geometry‐based respiratory‐motion‐simulating patient model for radiation dosimetry that uses Monte Carlo methods. The organ and body surfaces reconstructed using NURBS are shown in Fig. 3, which provides frontal and side views of skin, lungs, heart, rib cage, spine, and liver.

As described in the previous sections, we considered both a traditional conformal radiation treatment plan and an IMRT treatment plan for two delivery scenarios:

- “gating” treatment delivery, in which the radiation beam is turned on only when the patient's respiration reaches a particular breathing phase, and
- 4D image‐guided treatment delivery, in which the radiation beam follows the moving lesion in the respiratory cycle and is always turned on.

### A. Results for conformal radiation treatment plan

**Figure 4:**
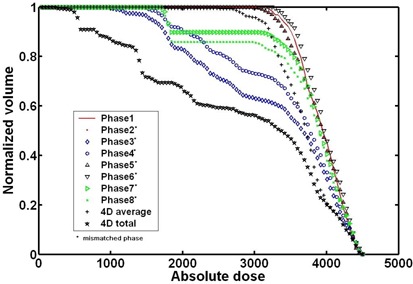
Dose–volume histogram plot for four‐dimensional (4D) simulation (phase 1, middle inhale; phase 2, late inhale; phase 3, peak inhale; phase 4, early exhale; phase 5, middle exhale; phase 6, late exhale; phase 7, peak exhale; phase 8, early inhale).

First, we compared both studied forms of treatment delivery for a conformal radiation treatment plan. Fig. 4 summarizes the dose–volume histograms (DVHs) delivered by the gating treatment and by the 4D image‐guided treatment for the lesion target. In the plan delivered using a gating method, the radiation beam is made to conform to the lesion according to its location in phase 1 (middle inhalation) of the respiratory cycle.

Fig. 4 shows that, the more similar the spatial and temporal distribution between a specific phase and phase 1 (during which the beam is aimed), the better the conformity of the DVH curve. Ideally, the beam is turned on only for phase 1. However, during gating treatment delivery, a phase may be mismatched by the gating window, and conformity suffers when that happens. Here, we simulated this scenario of a gating mismatch. Phases 1 to 8 were modeled as mismatched gating, and the results were averaged as the “4D‐average” by summing the doses in all individual phases and then dividing by the number of phases. The result represents the dose distribution when the gating is added in the whole respiratory cycle (Fig. 4). In contrast, the “4D‐total” DVH curve represents the photon beam planned for phase 1, but delivered in an ungated way. The result represents the dose distribution in the entire 4D free respiratory cycle.

Based on the DVH curves for the “4D‐average” and the “4D‐total,” it can be observed that, even though the gating technology may select a wrong phase to gate (4D‐average curve), the final result is still better than no gating at all (4D‐total curve). Therefore, the simulation results presented in Fig. 4 confirm that, as expected, the use of gating technology can improve the dose distribution.

### B. Results for IMRT plan

**Figure 5:**
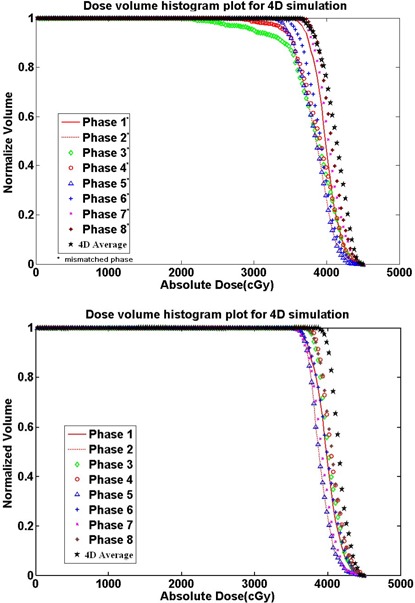
(a) Dose–volume histogram plot from intensity‐modulated radiotherapy (IMRT) gating simulation. (b) Dose– volume histogram plot results from IMRT four‐dimensional (4D) hybrid simulation.

Fig. 5(a) shows the DVH curves for the gated IMRT delivery. In that case, the ODM was aligned to the lesion, whose location is defined for phase 1, and the DVH for the 4D‐average showed better conformity than that for phase 1. The reason is the way in which the final dose for the IMRT plan is integrated. The data in Fig. 5 demonstrate that, in phases 3 and 4, the lesion is obviously underdosed because of respiratory motion; in other phases, the data exhibit varying degrees of discrepancy. The 4D‐average curve is derived by averaging the eight respiratory phases. The absolute dose of the 4D‐average at 90% is 3855 cGy.

Fig. 5(b) shows the DVH curves for the lesion at various phases for the 4D image‐guided treatment delivery. In that case, the center of the ODM is always conformal with the center of the lesion in each of the eight respiratory phases. This approach simulates an ideal 4D image‐guided treatment plan in which the beam moves accurately with the lesion through all eight respiratory phases. The data show an overall more uniform dose distribution for each of the eight phases. In addition, the averaged dose distribution (that is, 4D‐average) is still more uniform than the dose distribution of any single phase. The absolute dose of 4D‐average at 90% is 4060 cGy.

Comparing the DVH curves in Figs. 4 and 5, the varying effects of organ motion on the final dose distributions to the lesion can be observed. Figs. 4 and 5 both show that gating improves the dose distributions, even though a mismatched phase may occur [as in Fig. 5(a)]. For free‐breathing treatment, if the beam can be delivered to follow the breathing pattern, the 4D‐average curve in Fig. 5(b) suggests that the dose distributions would be better. However, if the beam fails to follow the breathing pattern, the dose distribution could be much worse, as shown in the 4D‐total curve in Fig. 4 discussed earlier. That finding confirms that, during 4D treatment delivery, monitoring of the patient's breathing pattern is critical. Technologies such as synchronized moving aperture radiation therapy (SMART) to improve monitoring accuracy have been reported.^(52)^

Planning and delivery of 4D radiation treatment is one of the most challenging tasks in the history of the discipline. A motion‐simulating model as reported in this study can be used to further investigate various issues in a controlled manner. For example, the aspect of 4D treatment delivery that raises difficulty is its reliance on more precise timing and positioning of the radiation beams for the increased conformity to the tumor of a 4D treatment plan over a 3D plan. Although approaches that use dynamic MLC (DMLC) for dose delivery have been proposed,^(^ ^15^ ^–^ ^17^ ^)^ more studies to demonstrate the efficacy of such methods are required before practical implementation can proceed. Image registration is generally based on either matching geometric image features or voxel similarity measures.^(^ ^53^ ^,^ ^54^ ^)^ Currently, studies about 4D image registration are based mainly on voxel similarity.^(^ ^55^ ^–^ ^61^ ^)^ However, geometric image features may be more useful for deriving information such as DVH curves, because the organ contours are available from the treatment planning system.

In the present study, we used surface equations to deform the organ contours, and we reversed the equations to obtain voxel‐to‐voxel integrated dose results. A deformable registration method, although not necessary for our study, was reported by Vedam et al.^(10)^ Tissue density change during 4D treatment has been discussed by Heath and Seuntjens.^(62)^ This latter issue is important for non‐rigid organs such as the lung, in which organ density changes from phase to phase. For tumors and organs that are more rigid, the density is assumed to be identical for the entire respiratory cycle. For non‐rigid organs, the DVHs may be derived from total energy deposited instead of from absorbed dose.

## IV. CONCLUSIONS

The present study demonstrates that many issues related to the management of respiratory motion could be investigated using a motion‐simulating, 4D anatomic and dosimetric model such as ours that is developed from the segmented Visible Human images and NURBS primitives. For our study, a lesion was modeled inside the lung to determine changes in dose distribution from a simplified external photon beam source. The results demonstrate the importance of using accurate “gating” to improve the dose distribution. The results also suggest that, during 4D image‐guided treatment delivery, monitoring of the patient's breathing pattern is critical and that accuracy‐improvement technologies such as SMART may be useful.^(52)^ Currently, we are refining our model and considering more dosimetry cases in the hope of providing insights into ways of refining strategies for the management of respiratory motion during radiation treatment. Our ultimate goal is to develop physics‐based, volume‐deformable models that take into account realistic human anatomy, physiology, and biomechanical tissue properties for organs of major interest in radiation treatment. A “virtual patient” of this kind can be expected to be a versatile test bed not only for advanced radiation treatment studies, but also for other biomedical studies that depend on a thorough understanding of the motions of interlinked organs in space and time.

## ACKNOWLEDGMENTS

The present project and previous work involving the original VIP‐Man model were funded through the following grants to Rensselaer: NSF CAREER/Biomedical Engineering BES‐9875532, NIH 1R03LM007964–01, and NIH 1R01CA116743–01. In addition, Dr. C.Y. Shi acknowledges grant support from the Cancer Therapy and Research Center: Cancer Center Council 10–000–022–459–08–849. Mr. J.Y. Zhang received a 2006–2007 Health Physics Society Graduate Fellowship. We acknowledge the helpful review comments made by our colleague at Rensselaer, Dr. Richard Radke.
